# Editorial: Year 2020: New Trends in Pharmacological Treatments for Osteoarthritis

**DOI:** 10.3389/fphar.2022.892934

**Published:** 2022-05-03

**Authors:** A. Fioravanti, S. Tenti, S. Cheleschi

**Affiliations:** Rheumatology Unit, Department of Medicine, Surgery and Neuroscience, Azienda Ospedaliera Universitaria Senese, Siena, Italy

**Keywords:** osteoarthritis, pharmacological treatment, chondrocytes, hyaluronic acid, disease modifying osteoarthritic drugs

## Introduction

Osteoarthritis (OA) is the most common rheumatic condition over the world and its prevalence is rising due to the increasing obesity and life span of the general population. It is estimated that the number of people affected by this condition will increase by approximately 50% over the next 20 years (Hunter and Bierma-Zeinstra, 2019; Long et al., 2022).

OA has a remarkable impact on functional ability and quality of life and it is one of the major causes of disability; the Global Burden of Diseases Study 2019 estimates that years lived with disability (YLD) due to OA increased of 114.5% from 1990 to 2019 (GBD 2019 Diseases and Injuries Collaborators, 2020). Unfortunately, its etiology is only partly understood and multiple factors ranging from aging to biomechanical stimuli contribute to the development and progression of the disease (Ren et al., 2020).

Growing evidence shows that OA is a complex condition, in which the whole joint is involved; degradation of articular cartilage, subchondral sclerosis, and hyperplasia of synovial tissue are hallmarks of OA (Goldring and Goldring, 2016). Destructive processes of articular cartilage play a pivotal role in the development and progression of the disease resulting from an imbalance between catabolic and anabolic events (Goldring and Goldring, 2016). However, the exact mechanism that drives OA is still poor understood, even if, it is assumed that different mediators contribute to the pathogenesis of the disorder (Cheleschi et al., 2018; Zheng et al., 2021); this circumstance poses a challenge for its management. In fact, current pharmacological treatments are mostly related to the relief of the symptoms, whereas disease-modifying OA drugs (DMOADs) (aimed at reducing symptoms in addition to slowing or stopping the disease progression) are not actually available (Latourte et al., 2020). The discovery of the crucial pathways characterizing OA could offer new opportunities to identify compounds potentially able to reduce or stop the disease progression (Latourte et al., 2020). The purpose of this Research Topic was to provide an overview of emerging preclinical (*in vivo*, animal models, and *in vitro*) and clinical studies testing different approaches for the treatment of OA.

## Overview of the Research Topic

The present special collection includes ten Original Articles, five narrative Reviews and one Systematic Review providing new insights on current and future therapeutic options for OA.

A large contribution has been provided on pre-clinical researches. In a study on human OA chondrocytes, Sanchez et al. investigated the effects of Zeel T (Ze14), a multicomponent medicinal product composed of plant and organ extracts, known for its symptomatic effects in observational clinical studies. The authors showed that Ze14 significantly inhibited cartilage degradation, reducing metalloproteinases expression, and promoted chondrogenesis (Sanchez et al.).

Along the same line, Baek et al. observed the protective effects of 3′-Sialyllactose, a compound derived of human milk, against the oxidative stress and inflammation processes induced by IL-1β, in SW1353 chondrocytic cells.

Selonsertib (Ser), an inhibitor of Apoptosis Signal-regulated kinase-1 (ASK1), has been studied both in *in vitro* and in *in vivo* models by Yan et al. The results of the study showed that Ser markedly prevented the IL-1β-induced inflammatory reaction, cartilage degradation and cell apoptosis in rat chondrocytes; besides, intra-articular (i.a.) injection of Ser, in rat OA model, significantly alleviated the progression of the disease (Yan et al.).

Furthermore, the pharmacological activity of seven commercially available mixtures of avocado/soybean unsaponifiables (ASUs) were studied by Lambert et al. on human OA chondrocytes cultured in alginate beads. The authors demonstrated the inhibitory effect of the mixture of PIASCLEDINE-ExpASU^®^ on pro-inflammatory and pro-catabolic factors.

Ma et al. observed that vanillic acid, a monomer derived from chinese herbal medicines, was able to target NLRP3 inflammasome reducing synovitis in a rat model of knee OA.

The unique contribution as systematic review and meta-analysis has been provided by Sumsuzzman et al., analyzing the available experimental data on animal models on the use of melatonin for the treatment of OA. The authors placing particular emphasis on the effects of exogenous melatonin in preventing OA pathogenesis through the regulation of circadian rhythms and anabolic/anticatabolic balance.

Furthermore, the review by Zhang et al. gives new information about the role of icariin in knee OA. Icariin is a flavonoid compound from the traditional Chinese medicine and it is known for its clinical efficacy in the treatment of bone and joint diseases. This review article confirms the potential role of icariin in alleviating knee OA, through the inhibition of inflammation, cartilage breakdown and extracellular matrix degradation.

One narrative review focused on recent developments of agents for the treatment of OA, providing a general overview on potential DMOADs, as lorecivivint, MIV-711 and spirifermin, and new therapeutic option for pain relief. Indeed, the most recent clinical trials and preclinical studies investigated a variety of possible therapeutics targeting of different underlying mechanisms, as inflammation, cellular senescence, cartilage metabolism, subchondral bone remodeling and peripheral nociceptive pathways. However, long-term randomized clinical trials are needed to confirm the safety and the efficacy of these novel pharmacological agents for OA (Cai et al.).

Another narrative review discussed the current evidence on the efficacy and safety of the i.a. therapy for thumb-base OA (TBOA). The authors presented literature data about i.a. corticosteroids, which remained a mainstay of therapy, i.a. hyaluronic acid and emerging i.a. agents, as platelet-rich plasma (PRP) or mesenchymal-derived stem cell populations. Despite, the i.a. therapy represents an attractive strategy for the local treatment of TBOA, within the multidisciplinary approach for the management of hand OA, the current evidence remains equivocal, mainly due to the heterogeneity among the conducted studies (Tenti et al.).

A mini review dealt with the articular and extra-articular effects of glucosamine sulfate in the treatment of OA. In particular, it was discussed the efficacy of glucosamine sulfate on OA pain and its potential structure-modifying effect in patients with knee OA. Furthermore, the authors focused on the protective role of glucosamine sulfate on the cardiovascular mortality, probably due to the modulation of the O-GlcNAcylation pathway (Conrozier and Lohse).

An opinion article by Scanu et al. provided a brief overview on the immunological events associated to OA and on the current and future therapeutics for OA. In addition, the authors described the role of balneotherapy in OA prevention and treatment with a particular highlight on the immunomodulatory properties of mineral waters.

Finally, Negrini et al. presented the case of two patients (one 85-year-old patient with severe functional impairment and one active 59-year-old patient) with knee OA treated with PRP injections, coupled with a post-treatment home-based rehabilitation program, consisting in a series of exercise to be performed at home, during the 5 days following PRP for two consecutive weeks. This therapeutic approach resulted safe and well tolerated and led to a significant improvement of pain and function, especially in the older patient. The authors concluded stating that the obtained results motivated them to plan further studies based on the same program, with the implementation of telemedicine and biomechanical evaluation to enhance compliance, efficacy, and outcomes.

The search for the pathological processes of bone formation and remodeling, and its implication in the pathogenesis of osteoporosis was performed in three original articles. Among them, Zhang et al. explored the role of Juglanin, a natural compound derived from the *crude Polygonum aviculare*, in RAW 246.7 macrophage cell line and in ovariectomized mice. The authors showed that Juglanin suppressed osteoclastogenesis inhibiting the expression of receptor activator of nuclear factor-κB ligand (RANKL) and NF-*κ*B signaling pathway (Zhang et al.).

In a similar manner, other authors (Sun et al., 2021) reported that total flavonoids of rhizoma drynariae promotes mineralization of bone graft and differentiation of osteoblasts in a dose-dependent manner in osteoblasts cultures and in Sprague-Dawley rats, partly related to the activation of Wnt/β-catenin signaling pathway.

The role of the insulicolide A, a natural nitrobenzoyl sesquiterpenoid derived from marine fungus, on RANKL stimulated osteoclastogenesis *in vitro* and on LPS induced osteolysis on mice model *in vivo* was investigated by Tan et al. The obtained results showed the inhibition of osteoclastogenesis and indicates that insulicolide A may have potential for the treatment of osteoclast related diseases such as osteoporosis or bone metastasis.

## Summary

Through this Research Topic efforts have been made in order to better understand the underlying pathophysiological mechanisms of OA and to investigate the potential effectiveness of a variety of natural and pharmacological agents. Furthermore, this special provides an update about some OA pathogenetic processes, as “anabolic and catabolic imbalance” and discusses the current evidence in the treatment of the disease, with a particular focus on i.a. therapy and glucosamine. This Research Topic demonstrates the growing interest in pre-clinical and clinical research in the field of OA, despite unmet needs remain in therapeutic area.
